# AICAR inhibits NFκB DNA binding independently of AMPK to attenuate LPS-triggered inflammatory responses in human macrophages

**DOI:** 10.1038/s41598-018-26102-3

**Published:** 2018-05-17

**Authors:** Johannes Kirchner, Bernhard Brüne, Dmitry Namgaladze

**Affiliations:** 1 Institute of Biochemistry I, Goethe-University Frankfurt, Theodor-Stern-Kai 7, 60596 Frankfurt, Germany; 2 Project Group Translational Medicine and Pharmacology TMP, Fraunhofer Institute for Molecular Biology and Applied Ecology IME, Theodor-Stern-Kai 7, 60596 Frankfurt, Germany

## Abstract

5-aminoimidazole-4-carboxamide-1-β-D-ribofuranoside (AICAR) is an established pharmacological activator of AMP-activated protein kinase (AMPK). Both, AICAR and AMPK were reported to attenuate inflammation. However, AICAR is known for many AMPK-independent effects, although the mechanisms remain incompletely understood. Here we report a potent suppression of lipopolysaccharide (LPS)-induced inflammatory gene expression by AICAR in primary human macrophages, which occurred independently of its conversion to AMPK-activating 5-aminoimidazole-4-carboxamide-1-β-D-ribofuranosyl monophosphate. Although AICAR did not interfere with activation of cytosolic signalling cascades and nuclear translocation of nuclear factor - κB (NFκB) by LPS, it prevented the recruitment of NFκB and RNA polymerase II to target gene promoters. AICAR also inhibited signal transducer and activator of transcription 3 (STAT3)-dependent induction of interleukin (IL) IL-6 and IL-10 targets, while leaving STAT6 and HIF1α-dependent gene expression in IL-4 and dimethyloxalylgylcine-treated macrophages intact. This points to a transcription factor-specific mode of action. Attenuated gene expression correlated with impaired NFκB and STAT3, but not HIF-binding in electrophoretic mobility shift assays *in vitro*. Conclusively, AICAR interferes with DNA binding of NFκB and STAT3 to modulate inflammatory responses.

## Introduction

AMP-activated protein kinase (AMPK) is a central metabolic regulator of eukaryotic cells^1,2^. Responding to increased AMP levels, AMPK inhibits anabolic biosynthetic pathways, while activating catabolic pathways, such as glycolysis, fatty acid oxidation, or autophagy. Additionally, in vascular or innate immune cells, AMPK attenuates inflammatory responses by interfering with nuclear factor - κB (NFκB) and c-Jun N-terminal kinase (JNK) pathways^3–6^. AMPK also modulates anti-inflammatory macrophage polarization by cytokines, such as interleukin-4 (IL-4) or IL-10 ^7–10^.

The ability of AMPK to interfere with a disturbed metabolism and chronic inflammation makes it an attractive pharmacological target for metabolic disease^11^. Therefore, much effort was undertaken to develop specific AMPK activators. 5-aminoimidazole-4-carboxamide-1-β-D-ribofuranoside (AICAR) was one of the first pharmacological AMPK activators^12^. Once it is taken up by the cell, AICAR undergoes phosphorylation by adenosine kinase to form 5-aminoimidazole-4-carboxamide-1-β-D-ribofuranosyl monophosphate (ZMP). ZMP binds AMPK at the AMP-binding site on the γ-regulatory subunit, causing allosteric activation of AMPK. Thereby AICAR promotes skeletal muscle glucose uptake^13^. Besides, AICAR causes a variety of AMPK-independent effects. Among them is inhibition of glycolysis and oxidative phosphorylation in the liver^14,15^, interference with cell cycle progression^16^, inhibition of dendritic cell maturation^17^, and induction of apoptosis^18^. Some of these effects are attributed to AICAR, and are potentiated by inhibiting the conversion of AICAR to ZMP. Mechanistically, AMPK-independent actions of AICAR are poorly understood and warrant further investigation.

Our previous work discovered AMPK-independent effects of AICAR on lipid metabolism and endoplasmic reticulum (ER) stress responses of human macrophages^19,20^. Conducting these studies we noticed that AICAR attenuated inflammatory responses of human macrophages to stimuli such as bacterial lipopolysaccharide. Similar observations have been reported, but how AICAR modulates inflammatory responses is still obscure^21–24^. We aimed to elucidate how AICAR interferes with LPS-induced inflammatory activation of human primary macrophages.

## Results

**Figure 1:**
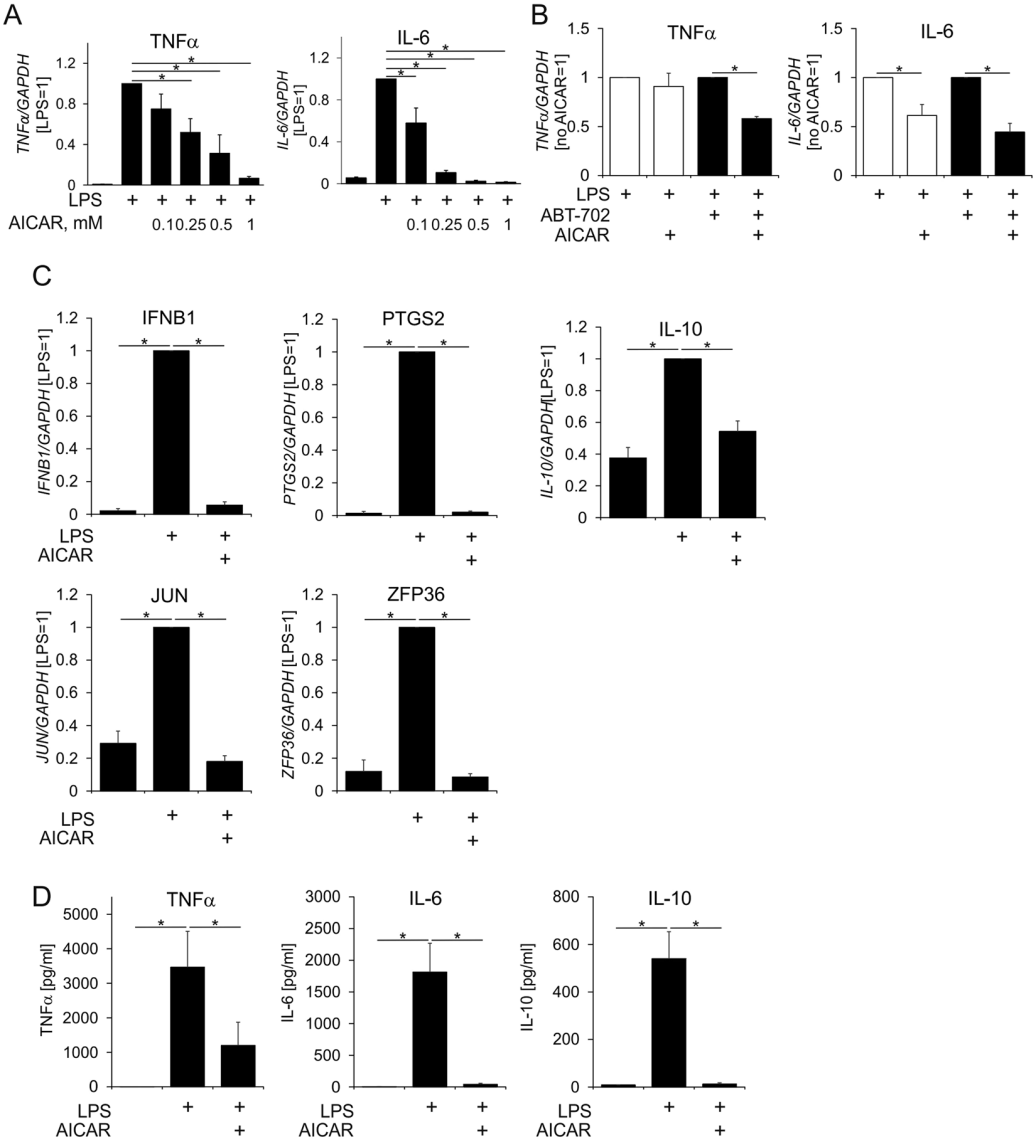
AICAR suppresses LPS transcriptional response. (**A**,**B**) mRNA expression of TNFα and IL-6 in macrophages treated for 3 h with 100 ng/ml LPS and indicated concentrations of AICAR (**A**) or with 100 ng/ml LPS, 0.1 mM AICAR and 0.5 µM ABT-702 (**B**). (**C**) mRNA expression of LPS-induced genes in macrophages treated for 1 h with 100 ng/ml LPS and 1 mM AICAR. (**D**) Cytokine secretion into culture medium of macrophages treated for 24 h with 100 ng/ml LPS and 1 mM AICAR. *p < 0.05.

To explore the anti-inflammatory mechanisms of AICAR we used primary human macrophages stimulated with LPS. In agreement with observations in murine macrophages^21^, AICAR, at concentrations shown to activate AMPK, inhibited typical LPS response genes, i.e. tumour necrosis factor α (TNFα) and IL-6 (Fig. 1A). Inhibition of adenosine kinase-mediated AICAR phosphorylation to ZMP, using the inhibitor ABT-702, left suppression of LPS-induced target genes by AICAR unaltered and even potentiated the effect of low AICAR concentrations (Fig. 1B), suggesting an AMPK-independent effect.

LPS induces an early and potent transcriptional response, which is largely dependent on the activity of NFκB and interferon response factor 3 (IRF3) transcription factors as well as the mitogen-activated protein kinase (MAPK) signalling cascade^25^. Analysing the mRNA expression of early induced genes targeted by NFκB/IRF3 (interferon beta 1 (IFNB1)), NFκB/MAPK (prostaglandin-endoperoxide synthase 2 (PTGS2)), or serum response factor (ZFP36 ring finger protein (ZFP36))^25^ as well as the AP-1 and NFκB target Jun proto-oncogene (JUN)^26^ 1 h after LPS treatment shows that AICAR inhibits the transcriptional response to LPS already at early times (Fig. 1C). AICAR also lowered transcriptional activation of an anti-inflammatory cytokine IL-10 by LPS (Fig. 1C). Blocking the LPS transcriptional response in the presence of AICAR strongly inhibited secretion of IL-6 and IL-10 into the culture medium of LPS-treated macrophages (Fig. 1D). Interestingly, TNFα secretion was only partly reduced by AICAR, which can be explained by the LPS-stimulated release of already pre-formed TNFα (Fig. 1D)^27^.

**Figure 2:**
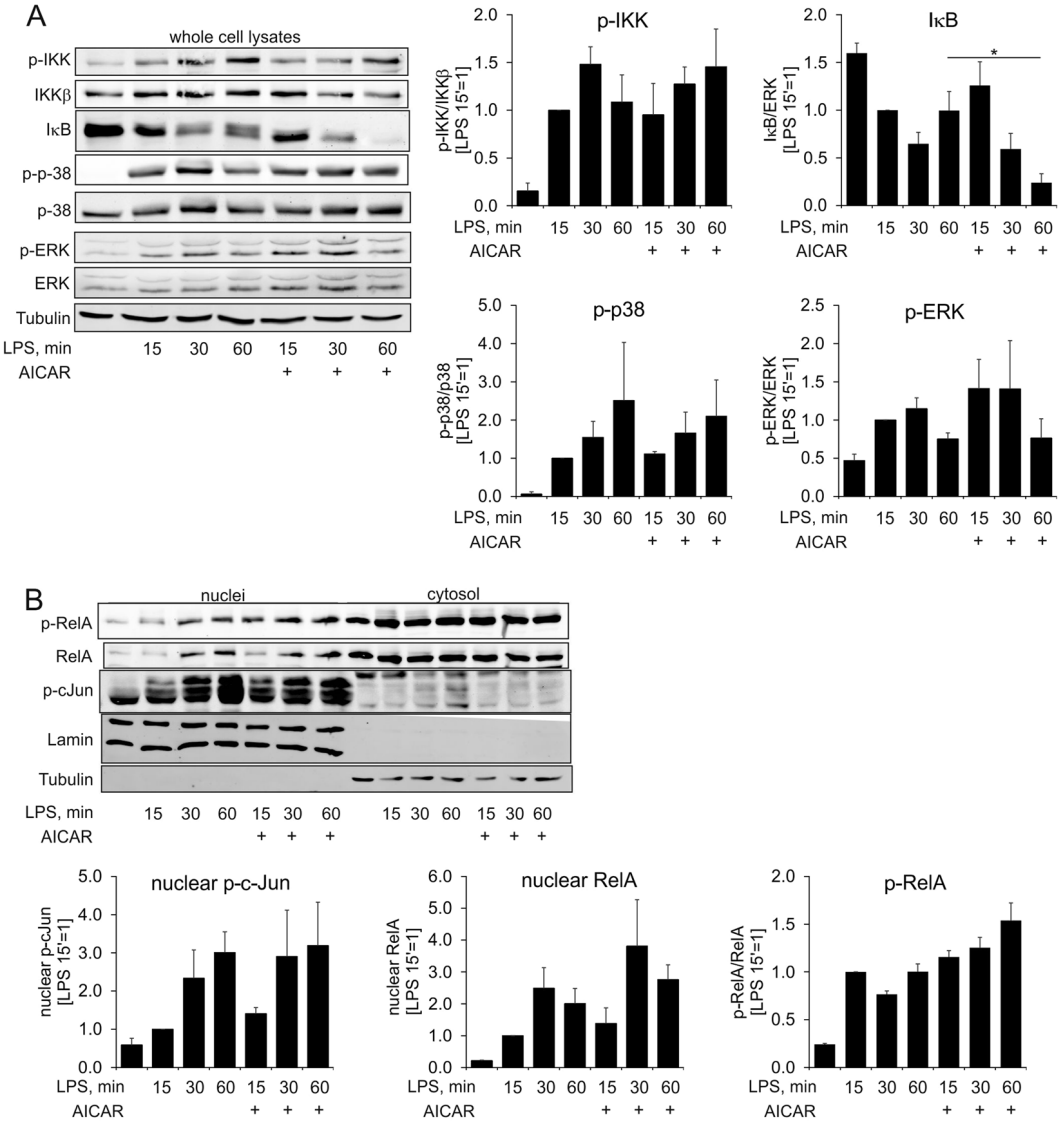
AICAR does not influence cytosolic LPS signaling. (**A**,**B**) Western blot analysis and its quantification (N = 5–6) for whole cell extracts (**A**) or nuclear fractions (**B**) of macrophages treated for indicated times with 100 ng/ml LPS and 1 mM AICAR. Full-length scans of the blots are presented in Supplementary Figure 1. *p < 0.05.

We went on to test the effect of AICAR on LPS-triggered cytosolic signalling cascades. AICAR did not influence early activation of the NFκB signalling pathway as seen by an unaltered IκB kinase (IKK) phosphorylation (Fig. 2A). IκB degradation was even more prominent in AICAR-treated cells (Fig. 2A). Following LPS stimulation, phosphorylation as well as nuclear translocation of the NFκB family member RelA (p65) also remained intact in the presence of AICAR (Fig. 2B). AICAR had also no effect towards activation of three major MAPK branches by LPS, since we observed similar phosphorylation of extracellular signal-regulated kinase (ERK), p38 MAPK, and nuclear c-Jun in macrophages stimulated with LPS in the presence or absence of AICAR (Fig. 2A,B).

**Figure 3:**
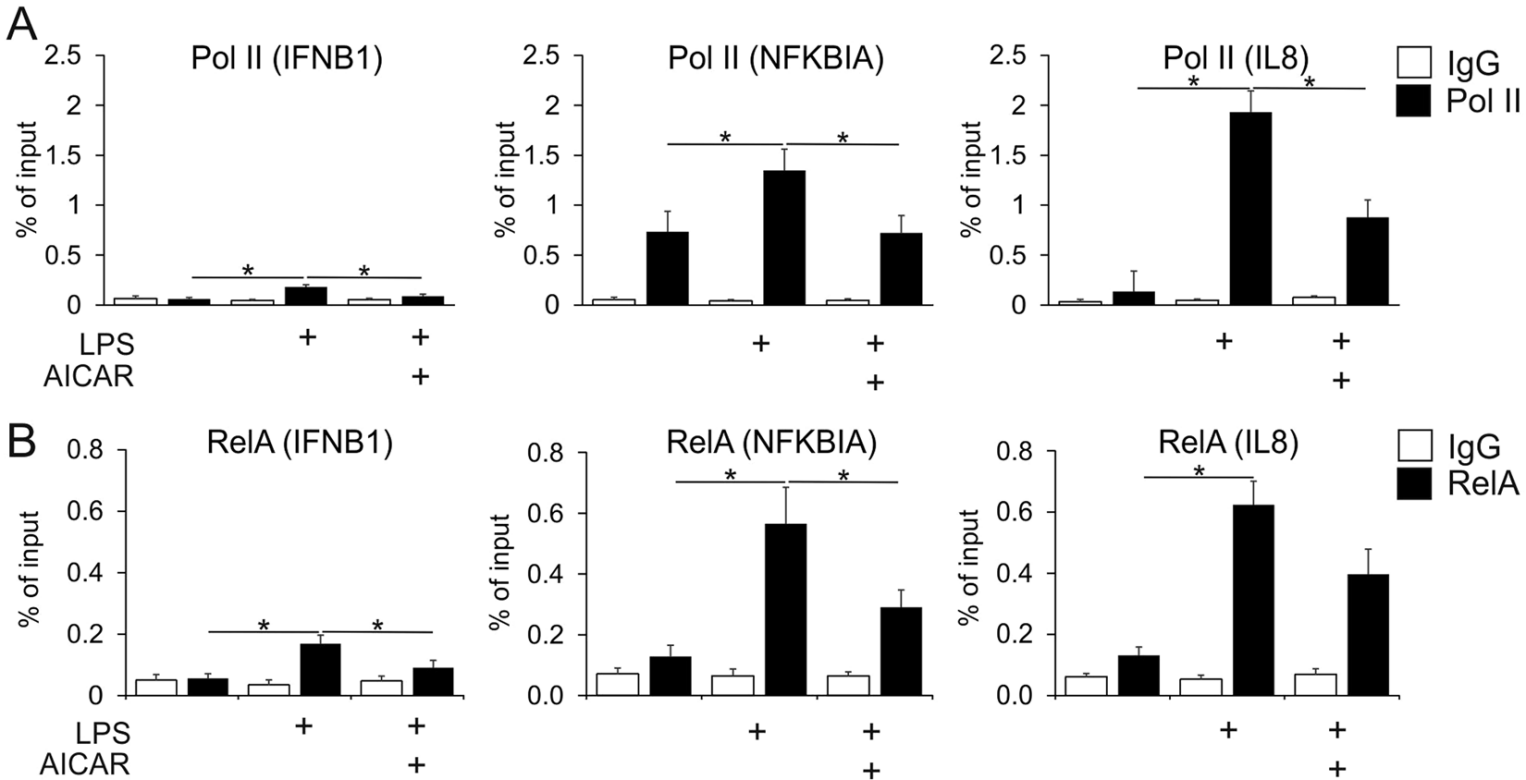
AICAR inhibits Pol II and RelA recruitment to promoters of LPS target genes. (**A**,**B**) Chromatin immunoprecipitation analysis using Pol II (**A**) or RelA (**B**) antibodies in macrophages treated for 1 h with 100 ng/ml LPS and 1 mM AICAR. *p < 0.05.

Genes activated by LPS differ in the degree of RNA polymerase II (Pol II) recruitment to their promoters. Some genes, such as IFNB1, have low levels of promoter-associated Pol II and require the recruitment of chromatin remodelling complexes for their activation. Others show substantial Pol II recruitment under resting conditions, being regulated at the transcriptional elongation phase^28^. To investigate whether AICAR interferes with transcriptional initiation triggered by LPS we performed chromatin immunoprecipitation (ChIP) experiments using antibodies against Pol II and RelA. We examined NFκB target genes regulated mostly by Pol II recruitment (IFNB1 and IL8) or transcriptional elongation (NFKBIA, encoding IκBα protein). Both IL-8 and NFKBIA mRNAs were rapidly induced by LPS in an AICAR-sensitive fashion (data not shown). Accordingly, ChIP experiments revealed low levels of promoter-associated Pol II at the IFNB1 and IL8 gene in untreated cells, whereas more Pol II was bound at the promoter of the NFKBIA gene (Fig. 3A). LPS increased Pol II recruitment to all target gene promoters, whereas AICAR attenuated this effect, consistent with the notion that AICAR interferes with the initiation phase of LPS-triggered transcription. ChIP analysis using the RelA antibody showed low levels of RelA binding to IFNB, IL8, and NFKBIA promoters in the basal state, whereas LPS treatment significantly increased RelA association with target gene promoters (Fig. 3B). Strikingly, RelA recruitment to target gene promoters was blocked when cells were treated with LPS in the presence of AICAR.

**Figure 4:**
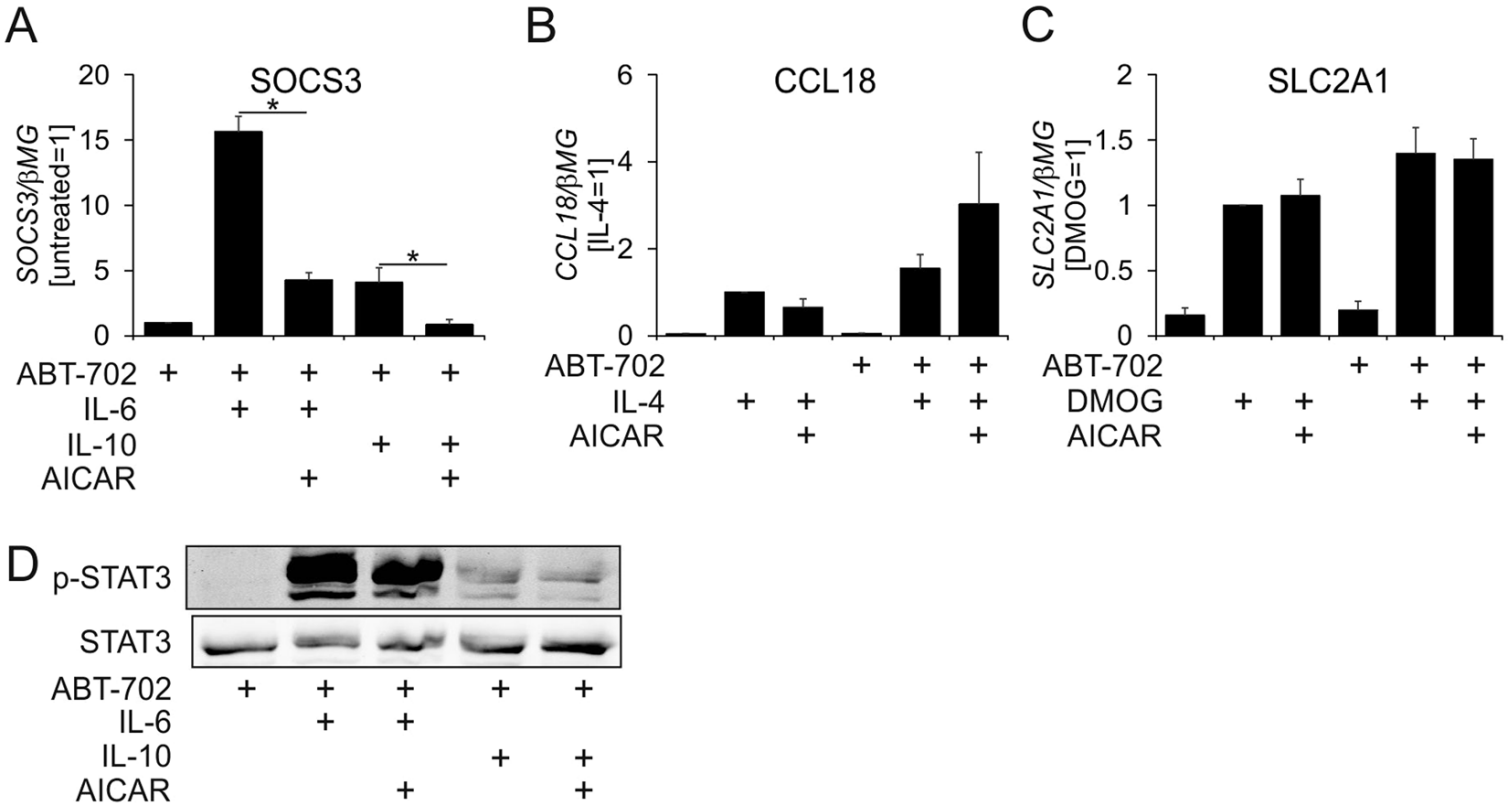
Stimulus specific regulation of transcriptional responses by AICAR. (**A**) mRNA Expression of SOCS3 in macrophages treated for 1 h with 20 ng/ml IL-6 or IL-10, 1 mM AICAR and 0.5 µM ABT-702. (**B**) mRNA Expression of CCL18 in macrophages treated for 24 h with 20 ng/ml IL-4, 1 mM AICAR and 0.5 µM ABT-702. (**C**) mRNA Expression of SLC2A1 in macrophages treated for 24 h with 1 mM DMOG, 1 mM AICAR, and 0.5 µM ABT-702. (**D**) Western blot analysis of STAT3 phosphorylation in macrophages treated for 1 h with 20 ng/ml IL-6 or IL-10, 1 mM AICAR and 0.5 µM ABT-702. *p < 0.05. Full-length scans of the blots are presented in Supplementary Figure 2.

Our current and previous findings indicate that AICAR prevents transcriptional activation by LPS or ER stress inducers without altering upstream signaling^20^. We considered that AICAR may interfere with a general component of the transcriptional activation machinery independently of the nature of a distinct transcription factor and activation stimulus. To test this hypothesis, we treated macrophages with agonists inducing well-characterized transcriptional responses. IL-4 predominantly acts via signal transducer and activator of transcription 6 (STAT6) transcription factor, while IL-6 and IL-10 induce target gene expression through STAT3. Transcriptional responses to hypoxia are mediated by hypoxia inducible factor 1α (HIF1α). HIF1α can be pharmacologically activated by the prolyl hydroxylase inhibitor dimethyloxalylglycine (DMOG). Since activated AMPK attenuates STAT3-dependent transcription induced by IL-6 or IL-4^9,29^, we blocked AICAR conversion to ZMP using ABT-702. Figure 4 shows that the ability of AICAR to inhibit activation of gene expression is stimulus-dependent. Whereas induction of STAT3-dependent SOCS3 mRNA by IL-6 or IL-10 was inhibited by AICAR in the presence of ABT-702 (Fig. 4A), IL-4-induced expression of the typical STAT6-dependent target gene CCL18 was unaltered (Fig. 4B). Similarly, HIF-dependent SLC2A1 mRNA expression was not affected by AICAR either in the absence, or in the presence of ABT-702 (Fig. 4C). AICAR did not inhibit cytosolic STAT3 activation after cytokine stimulation (Fig. 4D). Obviously, AICAR does not generally inhibit transcriptional activation, rather acting in a stimulus- and transcription factor-specific manner.

**Figure 5:**
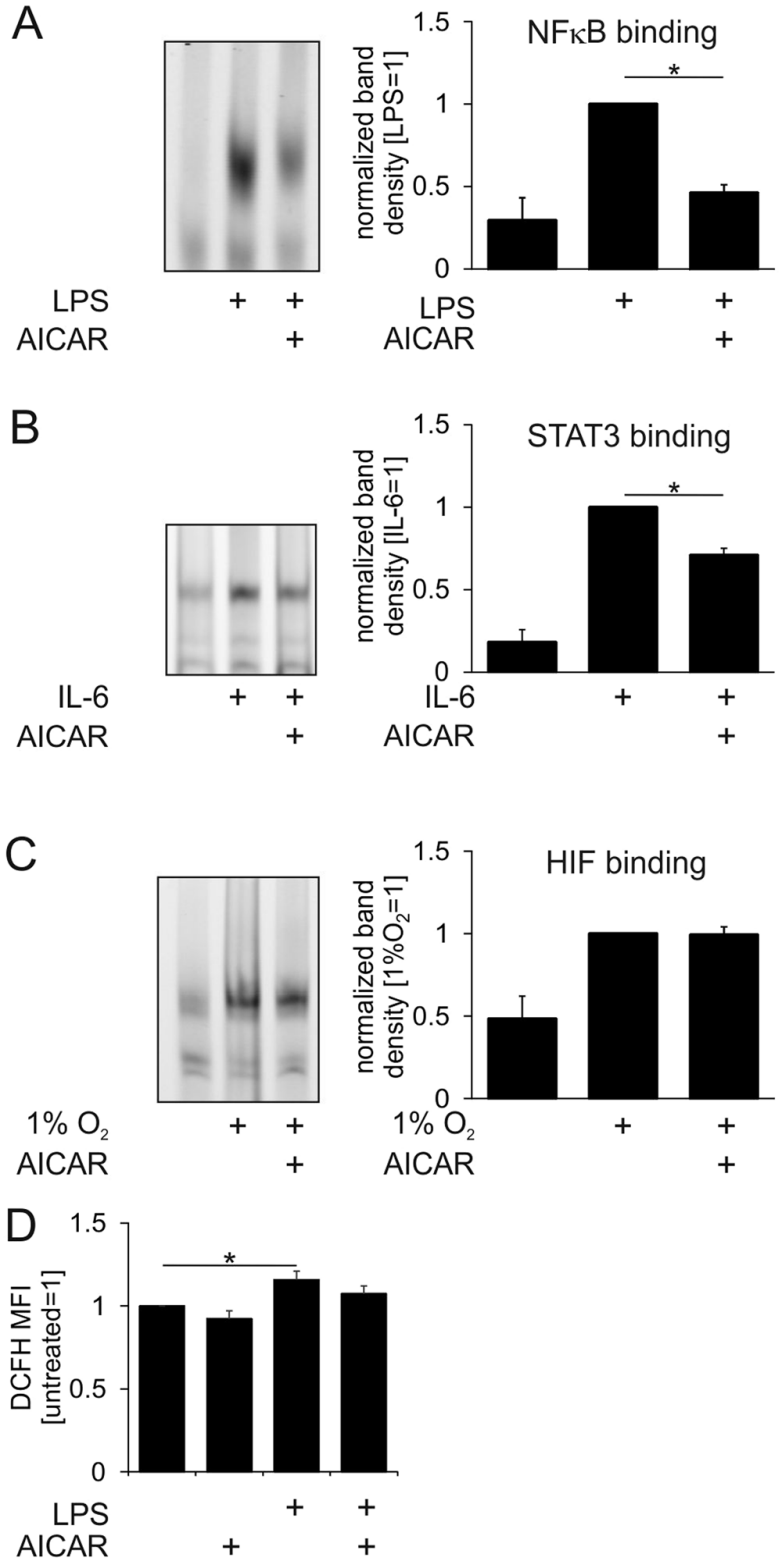
AICAR impairs transcription factor DNA binding *in vitro*. (**A**) EMSA of nuclear extracts of control and LPS-treated macrophages using a NFκB-specific oligonucleotide in the absence or presence of 1 mM AICAR. (**B**) EMSA of nuclear extracts of control and IL-6-treated macrophages using a STAT3-specific oligonucleotide in the absence or presence of 1 mM AICAR. (**C**) EMSA of nuclear extracts of macrophages cultured under normoxia or hypoxia (1% O_2_) using a HIF-specific oligonucleotide in the absence or presence of 1 mM AICAR. Full-length scans of the EMSA gels are presented in Supplementary Figure 3. **(D)** ROS levels in macrophages treated for 1 h with 100 ng/ml LPS and 1 mM AICAR. *p < 0.05.

Having shown transcription factor-specific inhibition of transcriptional activation by AICAR, we questioned the ability of AICAR to interfere with binding of transcription factors to DNA using electrophoretic mobility shift (EMSA) assays. Nuclear extracts were prepared from macrophages stimulated with LPS, IL-6 or cultured under hypoxia to achieve nuclear accumulation of NFκB, STAT3, or HIF1α, respectively. Subsequently, nuclear extracts were incubated with oligonucleotides containing NFκB, STAT3 and HIF1-binding sequences in the presence or absence of AICAR. Figure 5A–C shows increased oligonucleotide binding to stimulated nuclear extracts as compared to extracts prepared from untreated cells. Remarkably, incubations in the presence of AICAR attenuated NFκB and STAT3 DNA binding, whereas HIF-binding remained unaffected (Fig. 5A–C), correlating with the effects of AICAR on NFκB, STAT3, and HIF target gene expression in intact cells. Since NFκB p50 and p65 DNA binding is sensitive to oxidation of critical cysteine residues^30^, and AICAR may increase reactive oxygen species (ROS) in a cell type-specific manner^31^, we analysed intracellular ROS levels in LPS and AICAR-treated macrophages using 2′,7′-Dichlorodihydrofluorescein diacetate labelling and flow cytometry. As shown in Fig. 5D, LPS-treated cells exhibited slightly elevated ROS formation as expected. AICAR did not increase ROS levels either in the presence, or in the absence of LPS. These data suggest that a direct interference of AICAR with DNA binding rather than modulation of the redox environment explains inhibition of transcription factor activity.

## Discussion

A range of AMPK-dependent and -independent molecular effects have been ascribed to AICAR. Our data suggest that an interference with NFκB DNA binding explains a unexpectedly potent and broad anti-inflammatory action of AICAR in human macrophages. This effect occurs independently of AICAR conversion to ZMP and hence, AMPK activation. Our data contrast previous observations in murine macrophages linking inhibition of inflammatory responses by ACIAR to AMPK-dependent activation of the protein deacetylase Sirt1^21^. At the same time we corroborate findings of AMPK-independent anti-inflammatory effects in murine macrophage cell lines^23,24^. We show that anti-inflammatory effects of AICAR are relevant for the human innate immune system. AMPK-independent regulation of macrophage pro- vs. anti-inflammatory transcriptional responses by AICAR is not limited to NFκB-mediated transcription, but also extends to the cytokine-responsive transcription factor STAT3.

Strikingly, cytosolic LPS signalling is unaltered by AICAR under our conditions. The major outcome of cytosolic LPS-triggered signalling, nuclear translocation of transcriptional LPS effector RelA, remained intact in AICAR-treated cells as well. AICAR accumulation and conversion to ZMP is a relatively slow process, therefore, AMPK-dependent effects are less likely to take place during the initial phase of the LPS response, i.e. within 30 minutes of stimulation. This, as well as differences in cell types used may partly explain the discrepancies between our observations and reports showing that AICAR affects the activity of p38 and ERK signalling cascades in an AMPK-dependent manner^22,32^.

Although AICAR did not influence nuclear RelA accumulation in response to LPS, it blocked RelA binding to cognate DNA elements of LPS-induced genes as proven by ChIP assays. This may be sufficient to prevent transcriptional activation, since NFκB activation is a critical event during LPS transcriptional responses for most of its target genes^25^.

To explain the broad effect of AICAR on transcriptional activation we considered the interaction with general component of the transcription machinery. However, this is unlikely to be the case, since STAT6-dependent and HIF-evoked gene expression remained unaltered in AICAR-treated cells. AICAR also did not affect elevation of PPARγ mRNA expression during monocyte to macrophage differentiation^19^. At the same time, STAT3 target gene expression was inhibited by AICAR in AMPK-independent fashion. Previously, we showed broad inhibition of transcriptional unfolded protein response (UPR) by AICAR^20^, suggesting its direct interference with transcriptional UPR effectors, such as activating transcription factor 4 (ATF4), X-box binding protein 1, and ATF6. Thus, previous and current observations infer that transcriptional inhibition by AICAR applies to several transcription factors, at the same time excluding inhibition of the general transcription machinery.

EMSA experiments suggest a direct interference of AICAR with NFκB DNA binding and thus, NFκB-dependent transcriptional activation. Our data confirm previous reports showing that AICAR, when incubated with nuclear extracts from murine macrophages, directly interferes with DNA binding of NFκB, CREB and C/EBPβ^24^. In line, AICAR prevented DNA binding of NFκB and AP-1 in nuclear fractions of endothelial cells^33^. EMSA experiments conducted in AICAR-treated endothelial or tumour cells also showed decreased NFκB binding^34,35^, although under these conditions the effects were attributed to AMPK activation. Our data show that, in addition to NFκB, binding of STAT3 to its response element was also attenuated by AICAR. This correlated with inhibition of STAT3-dependent gene expression by AICAR. In contrast, neither HIF DNA binding, nor HIF-dependent transcriptional activation were inhibited by AICAR in macrophages. Collectively, our results indicate that the ability of AICAR to disrupt an interaction of a transcription factor with its DNA response element may account for the effect of AICAR on transcriptional activation. Structural determinants how AICAR interferes with DNA binding should be revealed in further experiments. High throughput DNA-binding assays may also identify the whole spectrum of transcription factor – DNA interactions sensitive to AICAR.

AICAR was tested in several clinical trials aiming to use it as an adenosine mimetic to ameliorate ischemia-reperfusion damage following coronary bypass surgery^36^. Our data point to potent anti-inflammatory effects of AICAR in combination with adenosine kinase inhibition. Although it is unlikely that this application of AICAR will find its way to the clinic, a mechanistic understanding how AICAR interferes with pro-inflammatory transcriptional activation may guide attempts to structurally alter this molecule to create optimized anti-inflammatory drugs.

## Methods

### Cell isolation and culture

Human peripheral blood mononuclear cells were isolated from buffy coats of anonymous donors (DRK-Blutspendedienst Baden-Württemberg-Hessen, Institut für Transfusionsmedizin und Immunhämatologie, Frankfurt, Germany) using Ficoll density centrifugation. Following 1 h culture in serum-free RPMI medium, non-adherent cells were removed by vigorous washing. Remaining adhered monocytes were differentiated into macrophages by 7 d culture in RPMI 1640 medium containing 3% AB-positive human serum. Cells were treated with the following reagents: 1 mM AICAR (EMD Biosciences), 0.5 µM ABT-702 (Tocris), 1 mM DMOG (Cayman), 100 ng/ml lipopolysaccharide (Sigma-Aldrich), 20 ng/ml IL-6, IL-10 or IL-4 (all Immunotools). Hypoxic incubations were performed using a hypoxic workstation with 1% O_2_, 94% N_2_, 5% CO_2_ at 37 °C (Invivo2 400, Ruskinn Technology).

Studies conform to the principles outlined in the Declaration of Helsinki and were approved by the ethics committee of the Faculty of Medicine at Goethe-University Frankfurt. The ethics committee waived the necessity of written informed consent when using the buffy coats from anonymized blood donors.

### Quantitative PCR

Total RNA of primary human macrophages was isolated using PeqGold RNAPure kit (PeqLab) and transcribed using cDNA Synthesis kit (Fermentas). Quantitative PCR was performed with iQ SYBR green Supermix (Bio-Rad) using the CFX96 system from Bio-Rad. Primer sequences for quantitative PCR can be obtained upon request. Expression was normalized to β-microglobulin.

### Western blot

Cell extracts were prepared using buffer (150 mM Tris-HCl pH 8, 150 mM NaCl, 5 mM EDTA,10 mM NaF, 1 mM Na_3_VO_4_, 0.5% NP-40, 2% sodium dodecyl sulfate, 10% glycerol, 10 mM DTT, 1 mM PMSF, protease inhibitor cocktail (Roche)). Nuclei were isolated using nuclear lysis buffer A (20 mM Tris-HCl pH 8.0, 10 mM NaCl, 5 mM EDTA, 0.5% NP-40, 1 mM PMSF, protease inhibitor cocktail) followed by centrifugation at 16000 g for 20 s. Nuclear pellets were sonicated in lysis buffer B (20 mM Tris-HCl pH 8.0, 400 mM NaCl, 5 mM EDTA, 0.5% NP-40, 1 mM PMSF, protease inhibitor cocktail). Protein lysates were separated on polyacrylamide gels followed by transfer onto nitrocellulose membranes. Membranes were incubated with antibodies against phospho-IKK (Ser176/Ser180) (#2697), phospho-p38 MAP kinase (Thr180/Tyr182) (#9211), phospho-ERK (Thr202/Tyr204) (#9101), phospho-cJun (Ser73) (#3270), IKKβ (#8943), ERK (#4696), p38 MAP kinase (#9212), RelA (#8242), phospho-RelA (Ser536) (#3033), IκB (#4814) (all Cell Signalling Technology) or nucleolin (sc-13057) (Santa Cruz Biotechnology) followed by IRDye 800-coupled secondary antibodies (LICOR Biosciences). Blots were visualized and quantified using the Odyssey imaging system (LICOR Biosciences).

### Chromatin Immunoprecipitation

10 × 10^6^ macrophages were crosslinked for 10 minutes with 1% formaldehyde. Nuclear extracts were sonicated to create DNA fragments of 300–800 bp size. Crosslinked chromatin was immunoprecipitated with 2.5 µg of Pol II antibody (sc-899X), RelA antibody (sc-109X. both Santa Cruz Biotechnology) or rabbit IgG overnight at 4 °C followed by incubation with Protein A/G Agarose beads (sc-2003, Santa Cruz Biotechnology) for an additional 2 h. Immunoprecipitated DNA was recovered using PCR purification kit (Qiagen) and analysed using quantitative PCR. Primers corresponding to human IFNβ promoter (5′-GGGAGAAGTGAAAGTGGGAAA-3′, 5′-CAGGAGAGCAATTTGGAGGA-3′) or IκB (5′-GACGACCCCAATTCAAATCG-3′, 5′-TCAGGCTCGGGGAATTTCC-3′) and IL-8 promoters (5′-GTTGTAGTATGCCCCTAAGAG-3′, 5′-GCCTTTGCATATATCAGACAG-3′) were used for analysis. Data are shown as percentage of input DNA precipitated by a corresponding antibody.

### Electrophoretic mobility shift assays (EMSA)

20 µg nuclear extracts from macrophages treated with 100 ng/ml LPS or 20 ng/ml IL-6 for 1 h or cultured under 1% O_2_ for 4 h were incubated for 15 min at room temperature in a mixture containing 20 mM HEPES, 70 mM KCl, 2 mM DTT, 4% Ficoll, 2% glycerol, 0.1 mg/ml poly-dIdC, and 12.5 nM IRDye700-labelled oligonucleotide (5′-AGTTGAGGGGACTTTCCCAGGC-3′ for NFκB, 5′- AGCTTCATTTCCCGTAAATCCCTA-3′ for STAT3, 5′- GCCCTACGTGCTGTCTCA-3′ for HIF). Samples were run for 1 h on 6% polyacrylamide gels at 80 V using USB Glycerol Tolerant Gel buffer (Affimetrix) and bands were visualized using Odyssey imaging system (LICOR Biosciences).

### Cytometric bead array assays

Accumulation of IL-6, TNFα, and IL-10 in culture medium of macrophages treated with for 24 h with 100 ng/ml LPS and 1 mM AICAR was analysed according to manufacturer’s instructions using cytometric bead array Flex Sets (BD Biosciences) on a LSRII/Fortessa flow cytometer and quantified using FCAP v3.0 (Soft Flow).

### ROS measurements

Macrophages were treated with 100 ng/ml LPS and 1 mM AICAR and labelled with 5 µM 2′,7′-dichlorodihydrofluorescein diacetate (Thermo Fisher Scientific) for the last 20 min of LPS treatment. Median fluorescence intensity (MFI) of the FITC channel was recorded on a LSRII/Fortessa flow cytometer.

### Statistical analysis

Data are presented as means ± SEM of at least three independent experiments. Data were analysed by one-way ANOVA with Bonferroni post hoc means comparison using Prism software (GraphPad). Differences were considered statistically significant for P < 0.05.

### Data availability

All datasets generated and analysed during the current study are present in the paper. Primary data sets are available from the corresponding author on reasonable request.

## Electronic supplementary material

Supplementary Information
