# Supplementary material for: AICAR inhibits NFκB DNA binding independently of AMPK to attenuate LPS-triggered inflammatory responses in human macrophages
Source: Sci Rep. 2018 May 17;8:7801. doi: 10.1038/s41598-018-26102-3 (PMC5958102; doi:10.1038/s41598-018-26102-3)
Supplement: Supplementary file 1 — Supplementary Information [file 41598_2018_26102_MOESM1_ESM.pdf]

AICAR inhibits NFκB DNA binding independently of AMPK to attenuate LPS-triggered inflammatory responses in human macrophages.

Johannes Kirchner, Bernhard Brüne, and Dmitry Namgaladze

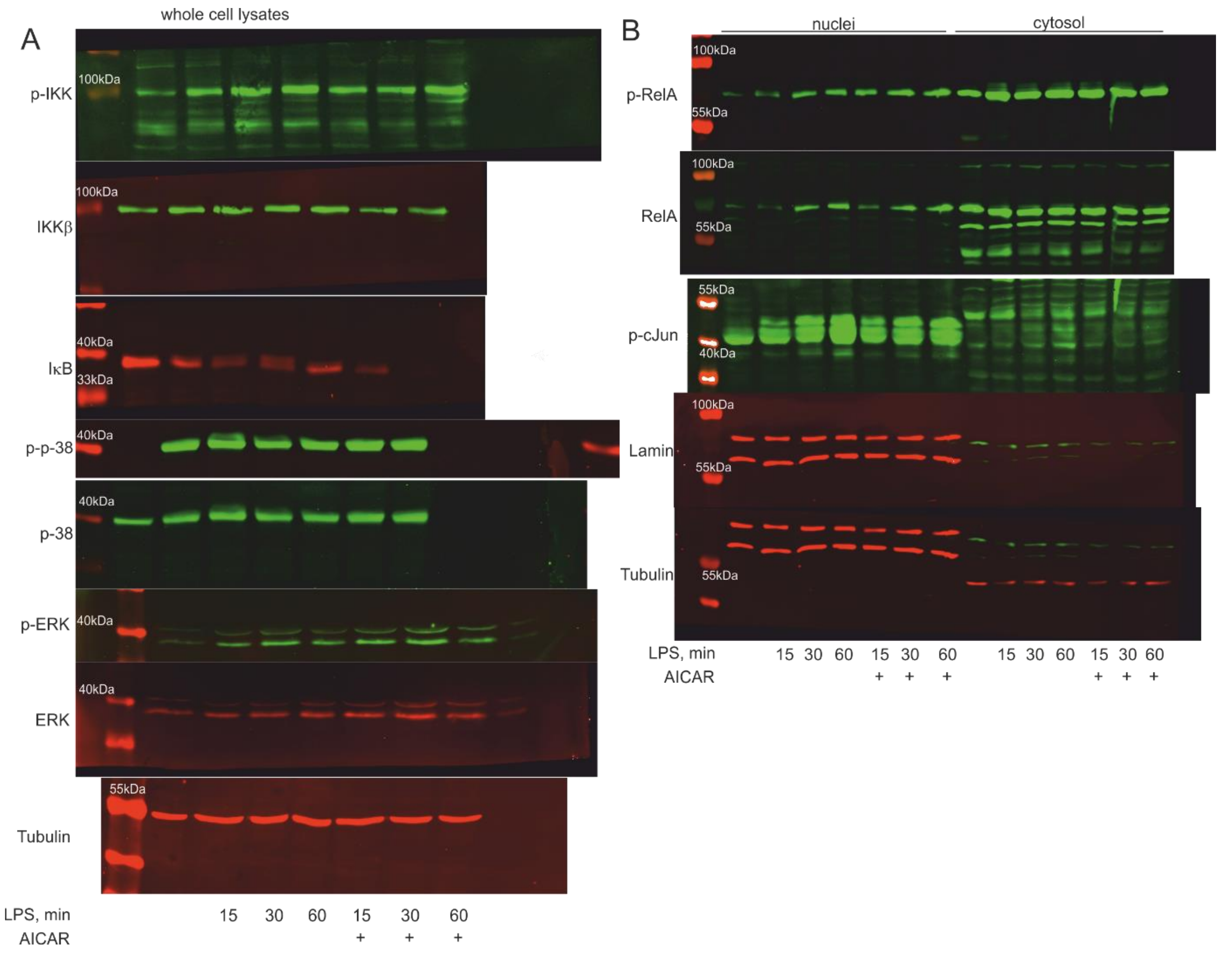

Supplementary Figure 1. Full-length scans of the blots presented in Figure 2.

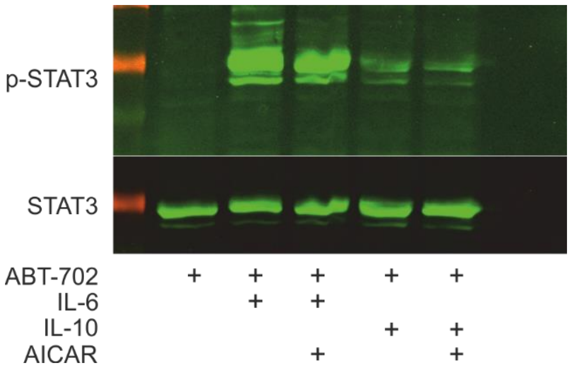

Supplementary Figure 2. Full-length scans of the blots presented in Figure 4.

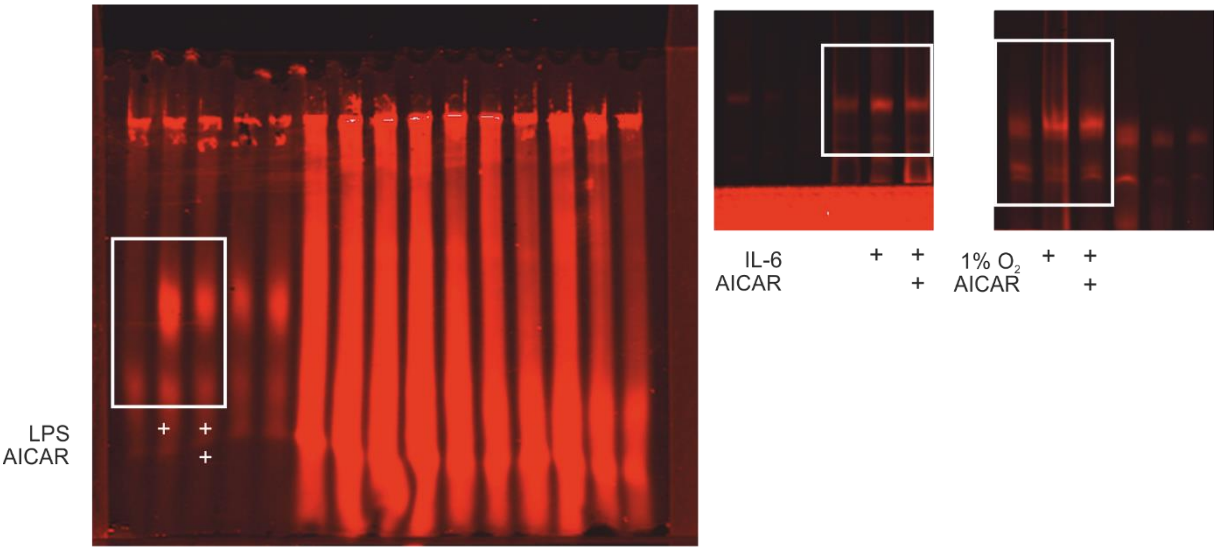

Supplementary Figure 3. Full-length scans of the EMSA gels presented in Figure 5.
